# Supplementary material for: Risk factors for Buruli ulcer disease in Ghana: A matched case-control study in four selected endemic districts of Eastern and Oti Regions
Source: PLoS Negl Trop Dis. 2025 Nov 11;19(11):e0013684. doi: 10.1371/journal.pntd.0013684 (PMC12604775; doi:10.1371/journal.pntd.0013684)
Supplement: S1 Table — (DOCX) [file pntd.0013684.s001.docx]

**S1 Table. Distribution of demographic and clinical characteristics by case classification**

| **Characteristic** | **Case classification** | | | | **Total n (%)** | |
| --- | --- | --- | --- | --- | --- | --- |
|  | **PCR-confirmed n (%)** | | **Probable n (%)** | |  |  |
| **Age group (years)** |  |  |  |  |  |  |
| 0–14 | 2 | (28.6) | 5 | (71.4) | 7 | (10) |
| 15–24 | 0 | (0.0) | 3 | (100.0) | 3 | (4.3) |
| ≥25 | 44 | (73.3) | 16 | (26.7) | 60 | (85.7) |
| **Sex** |  |  |  |  |  |  |
| Male | 27 | (64.3) | 15 | (35.7) | 42 | (60) |
| Female | 19 | (67.9) | 9 | (32.1) | 28 | (40) |
| **Religion** |  |  |  |  |  |  |
| Islam | 5 | (45.5) | 6 | (54.5) | 11 | (15.7) |
| Christianity | 41 | (69.5) | 18 | (30.5) | 59 | (84.3) |
| **Education** |  |  |  |  |  |  |
| No formal education | 11 | (73.3) | 4 | (26.7) | 15 | (21.4) |
| Primary | 24 | (66.7) | 12 | (33.3) | 36 | (51.4) |
| Secondary or higher | 11 | (57.9) | 8 | (42.1) | 19 | (27.1) |
| **Occupation** |  |  |  |  |  |  |
| Trader | 12 | (80.0) | 3 | (20.0) | 15 | (21.4) |
| Farmer | 23 | (76.7) | 7 | (23.3) | 30 | (42.9) |
| Student | 1 | (12.5) | 7 | (87.5) | 8 | (11.4) |
| Artisan | 2 | (66.7) | 1 | (33.3) | 3 | (4.3) |
| Fisher | 0 | (0.0) | 1 | (100.0) | 1 | (1.4) |
| Others | 8 | (61.5) | 5 | (38.5) | 13 | (18.6) |
| **Marital status** |  |  |  |  |  |  |
| Non-married | 24 | (58.5) | 17 | (41.5) | 41 | (58.6) |
| Married | 22 | (75.9) | 7 | (24.1) | 29 | (41.4) |
| **Household economic status** | | | | | | |
| Low | 19 | (70.4) | 8 | (29.6) | 27 | (38.6) |
| Average | 13 | (72.2) | 5 | (27.8) | 18 | (25.7) |
| High | 14 | (56.0) | 11 | (44.0) | 25 | (35.7) |
| **Clinical forms** |  |  |  |  |  |  |
| Nodule | 0 | (0.0) | 4 | (100.0) | 4 | (5.7) |
| Plaque | 1 | (100.0) | 0 | (0.0) | 1 | (1.4) |
| Oedema | 6 | (66.7) | 3 | (33.3) | 9 | (12.9) |
| Ulcer | 32 | (71.1) | 13 | (28.9) | 45 | (64.3) |
| Healed | 7 | (63.6) | 4 | (36.4) | 11 | (15.7) |
| **Lesion category** |  |  |  |  |  |  |
| Category I | 10 | (76.9) | 3 | (23.1) | 13 | (18.8) |
| Category II | 25 | (65.8) | 13 | (34.2) | 38 | (55.1) |
| Category III | 10 | (55.6) | 8 | (44.4) | 18 | (26.1) |
| **Location of lesion** |  |  |  |  |  |  |
| Lower limb | 44 | (65.7) | 23 | (34.3) | 67 | (95.7) |
| Upper limb | 2 | (66.7) | 1 | (33.3) | 3 | (4.3) |
