# Supplementary material for: Risk factors for Buruli ulcer disease in Ghana: A matched case-control study in four selected endemic districts of Eastern and Oti Regions
Source: PLoS Negl Trop Dis. 2025 Nov 11;19(11):e0013684. doi: 10.1371/journal.pntd.0013684 (PMC12604775; doi:10.1371/journal.pntd.0013684)
Supplement: S1 File — (PDF) [file pntd.0013684.s003.pdf]

Variable dictionary for dataset on Risk Factors for Buruli Ulcer Disease in Ghana: A Matched Case-Control Study in Four Selected Endemic Districts of Eastern and Oti Regions

Variable Name	Description	Response Options
**agegrp**	Age groups of the respondent	1 = 0–14<br>2 = 15–24<br>3 = ≥25
**sex**	Sex of the respondent	0 = Male<br>1 = Female
**relig_new**	Religious affiliation	0 = Islam<br>1 = Christianity
**edu_new**	Educational level	0 = No formal education<br>1 = Primary<br>2 = Secondary or higher
**occupation_new1**	Occupation	1 = Trader<br>2 = Farmer<br>3 = Student<br>4 = Artisan<br>5 = Fisher<br>6 = Others
**married**	Marital status	0 = Non married<br>1 = Married
**ses_status**	Household-economic status (quintiles)	1 = Low<br>2 = Average<br>3 = High
**radio to home_theatre**	Ownership of assets in the household	0 = No<br>1 = Yes
**Lesion_type***	Type of lesion observed	1 = Nodule<br>2 = Plaque<br>3 = Oedema<br>4 = Ulcer<br>5 = Healed
**Healed_duration**	Duration in months since lesion healing to recruitment	Range: 1 to 24 months
**cat***	Category of lesion	1 = Category I<br>2 = Category II<br>3 = Category III
**Location***	Lesion site	1 = Lower Limb<br>2 = Upper Limb
**Case_classification***	Case classification	1 = PCR-confirmed positive<br>2 = Probable
**Farming_1**	Engagement in farming	1 = No

Variable Name	Description	Response Options
		2 = Yes
**Farm_in_swampNew**	Farming in/near swamp	0 = No Farming<br>1 = Farming in Swamp<br>2 = Non Farming in Swamp
**Farm_close_riverNew**	Proximity of farm to river	0 = No Farming<br>1 = Farm close to river<br>2 = Non Farm close to river
**Protective_cloth**	Use of protective clothing during farming	0 = Do not Farm<br>1 = Farm with less protection<br>2 = Farm with more protection
**repellent_use**	Use of insect repellent or nets	0 = No<br>1 = Yes
**LLIN_usage_new**	Use of LLIN	0 = No<br>1 = Yes
**farm_cut**	Cuts experienced during farming	0 = Do not farm<br>1 = Farm without cut<br>2 = Farm with cut
**farm_bite**	Insect bites experienced during farming	0 = Do not farm<br>1 = Farm without bite<br>2 = Farm with bite
**plantation_new**	Plantation in immediate environment	0 = No<br>1 = Yes
**forest_imm_new**	Forest in immediate environment	0 = No<br>1 = Yes
**swamp_imm_new**	Swamp or wetland in environment	0 = No<br>1 = Yes
**river_imm_new**	River or stream in environment	0 = No<br>1 = Yes
**pipe_protect_well**	Use of piped or protected well	0 = No<br>1 = Yes
**unprotect_well**	Use of unprotected well	0 = No<br>1 = Yes
**river**	Use of river/stream as water source	0 = No<br>1 = Yes
**pond**	Use of pond, dugout, dam, or lake	0 = No<br>1 = Yes
**rain**	Use of rainwater	0 = No<br>1 = Yes
**sachet**	Use of sachet or bottled water	0 = No<br>1 = Yes
**drinking_source**	Main source of drinking water	0 = Improved<br>1 = Unimproved
**cooking_source**	Main source of water for cooking	0 = Improved

Variable Name	Description	Response Options
		1 = Unimproved
**bathing_source**	Main source of water for bathing	0 = Improved<br>1 = Unimproved
**washing_source**	Main source of water for washing	0 = Improved<br>1 = Unimproved
**wash_soap_new**	Use of local soap for washing	0 = No<br>1 = Yes
**BCG_scar_new**	Presence of BCG scar	0 = No<br>1 = Yes
**family_condition_new**	Family/close contact with BU-like lesions	0 = No<br>1 = Yes
**water_only**	Injury managed with water only	0 = No<br>1 = Yes
**water_and_soap**	Injury managed with water and soap	0 = No<br>1 = Yes
**water_and_salt**	Injury managed with water and salt	0 = No<br>1 = Yes
**alcohol**	Injury managed by rubbing with alcohol	0 = No<br>1 = Yes
**bandage**	Injury managed with adhesive bandage	0 = No<br>1 = Yes
**leaves**	Injury managed with leaves	0 = No<br>1 = Yes
**Outcome**	Case or control status	0 = Control<br>1 = Case
**group**	Respondent group identifier	Range: 1 to 70 (numeric, unique per group)
*data related to the case only		
