# Supplementary material for: Risk factors for Buruli ulcer disease in Ghana: A matched case-control study in four selected endemic districts of Eastern and Oti Regions
Source: PLoS Negl Trop Dis. 2025 Nov 11;19(11):e0013684. doi: 10.1371/journal.pntd.0013684 (PMC12604775; doi:10.1371/journal.pntd.0013684)
Supplement: S2 File — (DOCX) [file pntd.0013684.s004.docx]

**S2 File. Translation of the Abstract “Risk Factors for Buruli Ulcer Disease in Ghana: A Matched Case-Control Study in Four Selected Endemic Districts of Eastern and Oti Regions” into French**

L’ulcère de Buruli est une infection bactérienne provoquant des ulcères cutanés chroniques et pouvant entraîner une invalidité permanente en l’absence de traitement précoce. Malgré son impact sur la santé publique, son mode exact de transmission reste mal compris. Cette étude, menée dans quatre districts du Ghana où l’ulcère de Buruli est endémique, visait à mieux comprendre les comportements et facteurs environnementaux susceptibles d’exposer les populations à un risque d’infection. En adoptant une approche cas-témoins appariés selon un ratio de 1 : 2, l’étude a montré que les personnes exerçant des activités agricoles sans vêtements de protection adéquats et celles résidant à proximité des cours d’eau présentaient un risque accru de développer la maladie. À l’inverse, les personnes mariées et celles qui désinfectaient leurs plaies à l’alcool semblaient moins à risque d’être atteintes. Ces résultats étayent des stratégies préventives concrètes au niveau communautaire, telles que la promotion du port d’équipements de protection lors des activités agricoles et la sensibilisation à la prise en charge appropriée des blessures, susceptibles de contribuer à réduire le fardeau de l’ulcère de Buruli dans les zones d’endémie.
