# Supplementary material for: Risk factors for Buruli ulcer disease in Ghana: A matched case-control study in four selected endemic districts of Eastern and Oti Regions
Source: PLoS Negl Trop Dis. 2025 Nov 11;19(11):e0013684. doi: 10.1371/journal.pntd.0013684 (PMC12604775; doi:10.1371/journal.pntd.0013684)
Supplement: Attachment — Submitted filename: Reviewer comments_Dr MAMAN_Togo.docx [file pntd.0013684.s005.docx]

**Review**

**Introduction**

**Line 85** : Instead of the WHO by 16 countries, 14 of, state…………consider « 16 countries, of which 14 in the African region

**Lin 90** : In Ghana the first BUD case was reported since (year)

**Line 178** : A case of BUD is ………and/or confirmed by PCR (not sure understood, do you want to say that the case should always confirmed by the PCR ?? if so, then remove or)

**Line 243** : but for 3 on their uper limbs

**Line 250** : Of the 70 cases, 27 (38.6%) were documented as laboratory-confirmed Positive ; The other 43 (61.4%) were confirmed cliniclly ??? How are sure that they trully BU ?? The majority is in this group. If we consider a the case definition you provide in the methodology, They should not be consider as BU cases

**Line 252-255** : Table 1 : Laboratory outcome, please indicate the type of testing (PCR or Micorscopy)

45 had ulcer lesions (how many patients with ulcer were confirmed by PCR)

**Line 267 :** the married person are 29 (41.4%) How many of lab confirmed (PCR) patients are married ??

**Line 282-2024 :** Table 2

The breakdown of age showed that few participants were under 15 years. It’s know that this group of age is at high risk of BU in Africa. There so many person old than 25 years but it seems they would be more elder since the married persons account for 41%.

**Line 332-334 : Table 7** : For all analysis, the author should based the calculation only on those who were confirmed by PCR as BU cases even in univariate or in multivarialte analysis. This could show the exact figure of the relationship between the cases and control. Since the majority were not confirmed as BU cases as most of them had ulcer lesions which could be also counfused with other diseases.

The marital statut here is correlated with the high number of old person and who are also married and could not reflet the real association with the decrease of the risk of BU (Here again, how many married persons are positive for BU).

**Discussion**

**346-351** : This study has critical limitations as the author declares in the paper. The non availability of the PCR results could not let to consider the findings raised form this study. I think if it’s only the updating of the line liste of the patient, why the author didin’t until have all the results ?? If these results are avaiblable, it’s better to revise the list and re-analyse the data to hae a good idea and compare to the existing findings.
