# Supplementary material for: Risk factors for Buruli ulcer disease in Ghana: A matched case-control study in four selected endemic districts of Eastern and Oti Regions
Source: PLoS Negl Trop Dis. 2025 Nov 11;19(11):e0013684. doi: 10.1371/journal.pntd.0013684 (PMC12604775; doi:10.1371/journal.pntd.0013684)
Supplement: Attachment — Submitted filename: Response to Reviewers.docx [file pntd.0013684.s007.docx]

**Manuscript ID: PNTD-D-25-00993**

| **SN** | **Comments** | **Response** |
| --- | --- | --- |
|  | **Summary and General Comments** |  |
|  | **Introduction** |  |
| 1 | Line 78-79: modify to reflect the fact that permanent disfigurement and disability are likely the consequence of delayed treatment. | We have revised the sentence to clarify that permanent disfigurement and disability are most likely consequences of delayed treatment in line 79. |
| 2 | Line 133-134: only 81 cases reported in Ghana in 2023 yet authors had 70 cases in <1year period from 4 districts. Can the authors explain the discrepancy? | The apparent discrepancy arises from the case definition and recruitment period. Cases included in this study were identified through the national Buruli ulcer surveillance system between January 2018 and March 2022. As clarified in the updated case definition, both probable and confirmed cases were recruited. The 81 cases reported nationally in 2023 in the NBUCP Annual Report reflect surveillance data for that single year, whereas our dataset represents cumulative cases from four endemic districts over a multi-year period (2018–2022). |
| 3 | 1. Authors maintained Buruli Ulcer disease as BUD. However, the term “BU” has been used throughout portions of the manuscript but not defined. Is BU used in the same context as BUD by the authors? | In the manuscript, BU was intended to mean the same as BUD (Buruli ulcer disease). For clarity and consistency, we have revised the manuscript to use only BUD throughout and have removed instances of BU. |
| 4 | 2.The use of the phrase “since then” to precede lines 90-91 is misleading. This portrays the meaning Buruli ulcer cases are still on the rise in Ghana, even currently. Meanwhile the number of reported cases from Ghana have steadily been on the decline since 2019. In addition, the reference [15] used is outdated. | To avoid the misleading impression, we have expunged the phrase “since then” and revised the section accordingly. See lines 90-92. |
| 5 | It seems to suggest some sentences in the introduction are not complete, thus making it difficult to get the clear import of the message the authors are putting across. The first sentence on line 110 is incomplete. “Environmental factors are central”. It is not clear what the authors are saying by this sentence. Similarly, “Use of unimproved water sources (such as ponds and swamps) for bathing, washing, or drinking is strongly associated with BUD”. Do the authors by this sentence mean the risk of BUD infection or what? | We thank the reviewer for pointing out the lack of clarity. We have revised the section to complete the sentences and clarify the message. Specifically, we replaced “Environmental factors are central” with “Environmental factors play an important role in the transmission of BUD” [in line 107] and clarified that the use of unimproved water sources is strongly associated with increased risk of BUD infection in lines 110-112. |
|  | **Methods** |  |
| 6 | Did the authors include a time limit on the duration of BUD cases that were included in the study? | Yes please, a time frame was applied. Eligible cases were restricted to those identified between January 2018 and March 2022. We have revised the case definition in the methods section to clearly indicate this time limit in lines 176-179. |
| 7 | Was the map an original source document created by the authors? There is no mention of this or the copyright material under Figure 2. The authors need to indicate the source of the map, if generated by them, the software used and its copyright need to be included in the manuscript. | We thank the reviewer for this useful observation. The map in Figure 2 was created by the authors using ArcGIS 10.4. We have revised the figure legend to indicate that it is an original figure, and we have included the software used along with the relevant copyright statement in the manuscript. Please see lines 162-165. |
| 8 | On lines 156 -159, some percentages have been indicated and others in brackets. It is not clear what the authors intend to communicate by this, for instance “…main sources of water for drinking 20.9% (16.2% - 30.2%) and domestic activities 29.9% (26.5% - 37.8%) respectively” | Thank you for the observation. The percentages in brackets represent the minimum and maximum values across the four endemic districts. We have revised the text to clarify this by indicating them as ranges in lines 154-158. |
| 9 | It is interesting to note the numbers of BUD cases that were recruited in this study considering the study sites are not highly endemic districts and recruited cases were between July 2021 and March 2022. | We appreciate this observation. The study did not recruit cases only between July 2021 and March 2022. Rather, all cases from January 2018 to March 2022 in the selected districts were eligible to be included. We have revised the methods section to make this clearer in lines 179. |
| 10 | Authors should describe how samples were collected and transported for cases that were laboratory confirmed for BUD. How was IS2404 PCR performed for these patients? At least a reference should be provided for the diagnostic PCR method used | Thanks for the comment. All BUD cases were identified through the national surveillance system, with samples collected by trained healthcare workers, transported under cold chain, and confirmed using IS2404 PCR. References describing these procedures have been added to the methods section in lines 189-196. |
|  | **Ethics** |  |
| 11 | Authors need to indicate the age range they referred to as children in this study | We have revised the manuscript to indicate this age range in the methods section in line 202. |
| 12 | On line 197, authors indicated the “data was stored securely” but it is not mentioned where exactly this information was stored; device, cabinet etc. | We have revised the manuscript to include the fact that the information collected was stored on a password protected computer in line 210. |
|  | **Data analysis** |  |
| 13 | What is the origin of the STATA software on line 223? | Thanks for the observation. The orgin has been captured in the revised manuscript in lines 235 - 236. |
|  | **Results** |  |
| 14 | Titles of the Tables under the results section are long. Authors should revise the titles accordingly and exclude the addition of extraneous information such as “in the Akwapim South, Akwapim North-Okere, Jasikan and Biakoye Districts of Ghana; Community matched case control study, July 2021 to March 2022”. | We thank the reviewer for this suggestion. We have revised the table titles to make them concise by removing extraneous details such as study sites and period. |
| 15 | The median age reported in this study is far higher than what is known for cases from West Africa. Added to this is the fact that most lesions were located on the lower limbs (and were not PCR confirmed) which in this age group is more probable to be from other aetiologies such as venous ulcers. This should be acknowledged as a major limitation. | We acknowledge this limitation and have incorporated it into the manuscript. Specifically, we now state in lines 445-448: *‘Although most cases were PCR-confirmed positive, less than one-third were probable cases obtained through the national Buruli ulcer surveillance system. This inclusion may introduce uncertainty, as many of these probable cases had lesions on the lower limbs, which could also arise from other causes.’* |
| 16 | It is unclear how the household economic status was assessed and what the categorizations (low/average/ high) mean. This needs clarification in the methods section | The assessment of household economic status using Principal Component Analysis (PCA) and its categorisation into low, average, or high household economic status is detailed in the Data Management and Analysis section of the Methods. The PCA included 21 variables, and the Kaiser-Meyer-Olkin measure (0.624) and Bartlett test (p<0.001) confirmed the data were suitable for this analysis. Please see lines 238-247 for details. |
|  | **Discussion** |  |
| 17 | It is important for the authors to reorganize the discussion section to appropriately convey the findings of their study. Beyond a few sentences in the opening paragraph, there is a restatement of some results in paragraph 1. These should be discussed by comparing with previous studies and indicating the significance/ implications of the findings for BU control in Ghana and elsewhere. | We thank the reviewer for the comment. The discussion [in line 339 onwards] has been reorganised to compare findings with previous studies and highlight their implications for Buruli ulcer control. |
| 18 | Line 339-341: Which model are the authors referring to here. No such data are presented in the results section of the manuscript. It is unclear what variables were included etc. They cannot discuss results which have not been presented as it makes it unclear for readers and should be addressed. | We thank the reviewer for this observation. We have expunged the sentence referring to the model, since the data were not presented in the results section. |
| 19 | Line 346-369: These are limitations. It will be best to present these well after the study’s results and implications are discussed. | We agree with the suggestion of the reviewer. The limitations previously presented have been moved to the end of the discussion section in lines 438-461. |
| 20 | The authors acknowledge “recall bias “as a limitation of this study but failed to indicate the disease duration of participants that were recruited in this work. For those who had inactive BUD, what was the duration of illness before they were recruited in the study. This is a key confounder that can influence study results. | We acknowledge the concern regarding disease duration. Information on duration of illness for inactive BUD cases has been added to the results in lines 264-265 . For active BUD cases, while specific duration was not recorded, the data collection procedure minimised recall variability by instructing cases to report exposures in the year preceding onset and controls in the year preceding interview as stated in lines 228-230. |
| 21 | The authors indicated controls were selected from the homes or neighbouring communities of the cases. It seems rather interesting to read on line 367 the “geographic area for control selection was expanded”. Could the authors have introduced bias into the study by recruiting beyond the proposed sites initially indicated in the methods section? Kindly explain the nuances here. | We appreciate this comment. The phrase “expanded geographic area for control selection” may have been misleading. What we meant was that, in some cases, controls were recruited from slightly further away within the same community when immediate neighbours of cases were not eligible or available. We did not extend recruitment beyond the communities originally described in the methods. To avoid confusion, we have expunged this statement from the limitations section. |
| 22 | The authors mentioned the use of alcohol as a cleansing agent for the wounds. I am curious to know what type of alcohol they are referring to here | The alcohol referred to in the manuscript is methylated spirit, a common household alcohol-based disinfectant in Ghana, typically used as first-aid treatment for open wounds when one is hurt. This has been clarified in the revised discussion in line 414-415. |
|  | **Review** |  |
|  | **Introduction** |  |
| 23 | Line 85 : Instead of the WHO by 16 countries, 14 of, state…………consider « 16 countries, of which 14 in the African region | We thank the reviewer for this suggestion. The sentence has been revised in lines 84-86. |
| 24 | Lin 90 : In Ghana the first BUD case was reported since (year) | The sentence has been revised to specify the year the first case was reported in Ghana. Please see line 90. |
| 25 | Line 178 : A case of BUD is ………and/or confirmed by PCR (not sure understood, do you want to say that the case should always confirmed by the PCR ?? if so, then remove or) | Thank you for the comment. We agree that the initial phrasing was unclear. We have revised the case definition to separately describe probable and confirmed cases in lines 176-183. In this study, both probable cases and confirmed were included. This revision removes the ambiguity around the use of “and/or.” |
| 26 | Line 243 : but for 3 on their uper limbs | The sentence has been revised for clarity to read in lines 266-267: *“Almost all the cases [67 (95.7%)] had lesions on their lower limbs, while 3 (4.3%) had lesions on their upper limbs.”* |
| 27 | Line 250 : Of the 70 cases, 27 (38.6%) were documented as laboratory-confirmed Positive ; The other 43 (61.4%) were confirmed cliniclly ??? How are sure that they trully BU ?? The majority is in this group. If we consider a the case definition you provide in the methodology, They should not be consider as BU cases | We thank the reviewer for this important observation. Our updated case definition (in lines 176-183) clarifies that both probable and confirmed cases were included, consistent with the national Buruli ulcer surveillance system. We have acknowledged the inclusion of probable cases as a limitation in the discussion and have added a caution to readers to interpret the findings with this limitation in mind (please see lines 445-448). We also recommend that future studies in Ghana build on the design explored in this study but rely solely on PCR-confirmed cases to improve validity. |
| 28 | Line 252-255 : Table 1 : Laboratory outcome, please indicate the type of testing (PCR or Micorscopy) | Thanks for the observation. The type of testing has been indicated (which is PCR) in line 269. |
| 29 | 45 had ulcer lesions (how many patients with ulcer were confirmed by PCR) | We thank the reviewer for this important observation. Lines 262-264 now states: “*Of the 45 cases with ulcers, 32 (71.1%) were PCR-confirmed positive and the remaining 13 (28.9%) were probable cases*”. This breakdown has been included in the revised supplementary table (S1 table) and results section to provide clarity on the PCR confirmation status of ulcerative cases. |
| 30 | Line 267 : the married person are 29 (41.4%) How many of lab confirmed (PCR) patients are married ?? | Of the 29 married cases, 22 (75.9%) were PCR-confirmed positive and the remaining 7 (24.1%) were classified as probable cases. This information has been included in the revised supplementary table to clarify the PCR confirmation status among married cases. See lines 279-280. |
| 31 | Line 282-2024 : Table 2 The breakdown of age showed that few participants were under 15 years. It’s know that this group of age is at high risk of BU in Africa. There so many person old than 25 years but it seems they would be more elder since the married persons account for 41%. | We thank the reviewer for this observation. Indeed, only 7 (10%) of cases were under 15 years, consistent with national Buruli ulcer surveillance trends as reported in the 2024 Annual Report of the National Buruli Ulcer Control Programme, where this age group accounts for a smaller proportion of reported cases in some endemic districts despite being at higher risk. For instance, between 2018 and 2022, the proportion of cases under 15 years in Ghana ranged from 10.9% to 24.5% (2018: 18.8%, 2019: 19.2%, 2020: 10.9%, 2021: 24.59%, 2022: 20.8%), with a small fraction PCR-confirmed positive. In addition, most cases (60/70, 85.7%) were aged 25 years and above, with a median age of 50 years (IQR 3–91). When we sub-analyzed age by marital status, 31 (51.7%) of adults were not married while 29 (48.3%) were married, showing that many cases were adults but not necessarily older adults. |
| 32 | Line 332-334 : Table 7 : For all analysis, the author should based the calculation only on those who were confirmed by PCR as BU cases even in univariate or in multivarialte analysis. This could show the exact figure of the relationship between the cases and control. Since the majority were not confirmed as BU cases as most of them had ulcer lesions which could be also counfused with other diseases. | We thank the reviewer for this important suggestion. At the time of initial data extraction, some laboratory results had not been updated in the line-list. After thorough reconciliation with reference laboratory records, 19 of the 43 cases initially classified as 'non-positive' were PCR-confirmed positive and have been updated in the revised results. The updated case definition (in lines 176-183) in the methods section now clarifies that both probable and confirmed cases were included.  We acknowledge that including probable cases may affect the precision of associations in both univariate and multivariate analyses. A sub-analysis restricted to PCR-confirmed cases would provide exact estimates of the relationship between cases and controls; however, reducing the number of cases from 70 would limit statistical power and could compromise the ability to detect meaningful associations. In this regards, readers have been cautioned in the limitation (in lines 438-439) to interpret the findings with this consideration in mind. Nonetheless, we recommend that future studies in Ghana build on the current study design but rely solely on PCR-confirmed cases. This would provide more precise estimates of associations between exposure variables and Buruli ulcer and improve the validity of both univariate and multivariate results. |
| 33 | The marital statut here is correlated with the high number of old person and who are also married and could not reflet the real association with the decrease of the risk of BU (Here again, how many married persons are positive for BU). | We agree with the reviewer that marital status is correlated with older age and that this could influence the observed association with BUD. Of the 29 married cases, 22 (75.9%) were PCR-confirmed positive. Age, sex, and community were matched during the study design. After multivariable analysis, being married remained associated with lower odds of BUD (aOR 0.32, 95% CI 0.13–0.78). Variables such as including educational level were not statistically significant. While these findings suggest a protective effect, residual confounding cannot be completely excluded. This has been noted in lines 364-366. |
|  | **Discussion** |  |
| 34 | 346-351 : This study has critical limitations as the author declares in the paper. The non availability of the PCR results could not let to consider the findings raised form this study. I think if it’s only the updating of the line liste of the patient, why the author didin’t until have all the results ?? If these results are avaiblable, it’s better to revise the list and re-analyse the data to hae a good idea and compare to the existing findings. | We thank the reviewer for this important suggestion. We would like to highlight that this study relied on existing cases already identified through the national Buruli ulcer surveillance system. Following the reviewer’s suggestion, the line-list was thoroughly reconciled with reference laboratory records, and the results were updated accordingly, identifying additional PCR-confirmed positive cases. The final analysis includes both PCR-confirmed and probable cases, consistent with the national surveillance system. While the estimated sample size of 70 cases was based on this inclusive definition, we acknowledge in the limitations that including probable cases may affect the precision of findings and readers are cautioned to interpret the findings with this consideration in mind (see lines 445-448). |
