# Supplementary material for: Risk factors for Buruli ulcer disease in Ghana: A matched case-control study in four selected endemic districts of Eastern and Oti Regions
Source: PLoS Negl Trop Dis. 2025 Nov 11;19(11):e0013684. doi: 10.1371/journal.pntd.0013684 (PMC12604775; doi:10.1371/journal.pntd.0013684)
Supplement: Attachment — Submitted filename: Response_to_Reviewers_auresp_2.docx [file pntd.0013684.s008.docx]

| **SN** | **Comments** | **Response** |
| --- | --- | --- |
| 1 | In Table 3, What is meant by ‘farm proximal to water bodies’ and ‘farm distal to water bodies’. What do ‘proximal’ and ‘distal’ imply in this situation? | The terms *“farm proximal to water bodies”* and *“farm distal to water bodies”* referred to whether participants farmed *near* or *away from* rivers, streams, or swamps, respectively. These terms have been modified in the revised version for clarity. Please see **line 305**. |
| 2 | Line 479-480: Conclusion: As stated in the results section, farming close/ near water bodies was the identified risk factor (not living near water bodies). This should be corrected appropriately. | Thank you for the comment. Both *farming close to waterbodies* and *presence of waterbodies in the immediate environment* of an individual were significant at *p* < 0.05 in the univariate analysis. As indicated in the manuscript, *farming close to waterbodies* was excluded from the final multivariable model due to collinearity with other farming-related variables. This information, along with additional details, is noted in the footnote of Table 7 (Please see **line 337**).  *Presence of waterbodies in the immediate environment* remained significant at *p* < 0.05 in the conditional multivariable model and therefore represents the true independent risk factor for BUD. The conclusion is consistent with this final model finding. |
| 3 | Thank you for stating "Participation in the study was entirely voluntary, and informed consent was obtained from all respondents. For child participants less than 18 years, written informed consent was obtained from a parent or guardian prior to inclusion"  Please specifically state whether written or verbal consent was obtained from the Adult Respondents. | The section has been revised to specify that written informed consent was obtained from all adult respondents. Please see **line 201**. |

**Manuscript ID: PNTD-D-25-00993R2**
